# Zika virus congenital syndrome: experimental models and clinical aspects

**DOI:** 10.1186/s40409-017-0131-x

**Published:** 2017-09-15

**Authors:** Carolina Manganeli Polonio, Carla Longo de Freitas, Nagela Ghabdan Zanluqui, Jean Pierre Schatzmann Peron

**Affiliations:** Neuroimmune Interactions Laboratory, Immunology Department – ICB IV, University of São Paulo (USP), Av. Prof. Lineu Prestes, 1730, Cidade Universitária, São Paulo, SP CEP 05508-900 Brazil

**Keywords:** Zika virus, Congenital infection, Arthrogryposis, Ocular abnormality, Experimental models

## Abstract

Viral infections have long been the cause of severe diseases to humans, increasing morbidity and mortality rates worldwide, either in rich or poor countries. Yellow fever virus, H1N1 virus, HIV, dengue virus, hepatitis B and C are well known threats to human health, being responsible for many million deaths annually, associated to a huge economic and social cost. In this context, a recently introduced flavivirus in South America, called Zika virus (ZIKV), led the WHO to declare in February 1st 2016 a warning on Public Health Emergency of International Concern (PHEIC). ZIKV is an arbovirus of the *Flaviviridae* family firstly isolated from sentinels *Rhesus* sp. monkeys at the Ziika forest in Uganda, Africa, in 1947. Lately, the virus has well adapted to the worldwide spread *Aedes aegypti* mosquito, the vector for DENV, CHIKV, YFV and many others. At first, it was not considered a threat to human health, but everything changed when a skyrocketing number of babies born with microcephaly and adults with Guillain-Barré syndrome were reported, mainly in northeastern Brazil. It is now well established that the virus is responsible for the so called congenital Zika syndrome (CZS), whose most dramatic features are microcephaly, arthrogryposis and ocular damage. Thus, in this review, we provide a brief discussion of these main clinical aspects of the CZS, correlating them with the experimental animal models described so far.

## Background

Since the first semester of 2015, Brazil has experienced some unprecedented epidemics of babies born with microcephaly as well as of adults with peripheral flaccid paralysis, suggestive of Guillain-Barré syndrome (GBS). The first cases were detected in the states of Bahia, Pernambuco and Paraíba, which are still now the epicenter of the crisis. Although many possibilities were considered, it is now well established that both are caused by a recently introduced virus named Zika virus (ZIKV) [1–4].

The ZIKV is an arbovirus that belongs to the *Flaviviridae* family. It was first isolated from sentinels *Rhesus* sp. monkeys from the Ziika forest in Uganda, Africa, in 1947 [5]. Further, the virus was also isolated from the sylvatic vector, a mosquito of *Aedes africanus* species. Interestingly, the virus has well adapted to other species of mosquitoes, but most importantly to *Aedes aegypti* [6], widely spread through the globe, and a well-known vector for many other viruses, including dengue (DENV), West Nile (WNV), yellow fever (YFV), chikungunya (CHV), Japanese encephalitis (JEV) and many others [7, 8].

ZIKV genome is composed of a positive single-stranded RNA that codifies three structural proteins, capside (C), pre-membrane (Pr-M) and envelope (Env), and seven non-structural proteins, NS1, NS2a-2b, NS3, NS4a-4b and NS5 [9]. The biological function of these proteins, either in the invertebrate or in vertebrate hosts have just started to be elucidated. Whereas structural proteins are important molecules for cell invasion [10–12], and to induce the immune response, non-structural proteins are important for viral replication and immune response evasion [13, 14]. Recently, it was shown that ZIKV infectivity of *Aedes aegypti* depends on NS1 antigenemia. Interestingly, the Asian strain is much more infectious to mosquitoes than the African strain, mainly due to an alanine-to-valine amino acid substitution at the residue 188 of the NS1 [15].

Although ZIKV is considered an emerging infectious disease, it has been neglected for many years since human infection was early reported in Africa and Asia [9]. Initially, it was not considered a threat to human health, as the infection was considered mild and benign. In 2007, at the Yap Island, Micronesia, ZIKV infection started to reach a wider and more pronounced spectrum. A large number of people presented symptoms like moderate fever (37.8 to 39.5 °C), headaches, arthralgia on hands and feet, conjunctivitis and cutaneous rash. These patients were erroneously diagnosed with DENV, but soon later it was discovered to be the first ZIKV outbreak in history [16]. Thus, differential signs and symptoms of each patient must be evaluated cautiously, as due to the similarity with other *Flavivirus* infections such as dengue and chikungunya, ZIKV infection may be misdiagnosed. A summarized list of differential diagnosis, laboratory tests and patient managing protocols have been elaborated [17].

Further ZIKV outbreaks have been reported in many countries. The first one happened in French Polynesia in 2014, with more than 28,000 people infected [18]. Outbreaks were also reported in Tahiti [19] and New Caledonia [20]. In fact, ZIKV has been recently found in 69 countries worldwide, according to the World Health Organization (WHO) [21]. What is worthy to mention is the fact that, although a great number of people was infected by ZIKV in French Polynesia, at that time, no increase in the rates of babies born with microcephaly, neither in adults with GBS was reported. Interestingly, however, a retrospective analysis revealed that indeed there was a significant increase in the microcephaly rate per live births in French Polinesia in 2014 [22].

The relevance of ZIKV infection during pregnancy has gained great notoriety since the huge increase in the number of babies born with microcephaly, especially in the northeast of Brazil. Because of that, on February 1st 2016 the WHO declared a Public Health Emergency of International Concern (PHEIC). Nowadays, according to the Brazilian Ministry of Health, there are 2660 babies born with microcephaly caused by ZIKV, and yet about 6000 cases have to be confirmed. Bahia (433 cases), Pernambuco (408 cases) and Paraíba (191 cases) states are the most affected [23].

It is now well accepted that microcephaly is only one of the features of the so-called congenital Zika syndrome (CZS). Despite being unquestionably the most dramatic, infants may also display several other problems, such as arthrogryposis, intrauterine growth restriction (IURG), uveitis and retinal degeneration [3, 24]. In fact, it has been recently shown that even babies born without microcephaly may display serious brain lesions [24].

## Zika virus and microcephaly

Since the second semester of 2015, due to the increased incidence of microcephaly in Brazil, many researchers and physicians suggested a causal relation between them, although there were no clinical or experimental evidences to support this statement. One of the first strong evidence of causal relation between ZIKV and microcephaly was reported in March, 2016. A woman working as a volunteer in Natal, capital of Rio Grande do Norte state in Brazil, had become pregnant in February, 2015. At the 13th gestational week, she had high fever, severe musculoskeletal and retroocular pain and maculopapular rash. Exams performed until 20th gestational week revealed no fetal alterations. Nevertheless, at 29 weeks of gestation, the patient returned to Europe, and examinations revealed the first signs of fetal damage, which was confirmed at 32 weeks. Ultrasound showed IURG associated with placental-artery calcification. Brain imaging evidenced cortical and subcortical calcifications with moderate ventriculomegaly, smaller cerebellum and brain stem, resulting in a head circumference below 26 cm, which indicates microcephaly. Due to the high degree of fetal damage, the decision on interrupting the pregnancy was made.

*Post-mortem* evaluation of fetal tissue revealed PCR positive for ZIKV whereas negative to other flavivirus (DENV, YFV, WNV and tick-borne encephalitis virus). Complete ZIKV genome was recovered from the brain tissue with 99.7% identity to ZIKV Asian strain originated from French Polynesia, which happens to be the strain currently circulating in Brazil. Anatomical and histological analyses showed loss of gyration of the cortex, left ventricle collapsed and right ventricle dilated. This was associated with astroglyosis in the subarachnoid space, slight infiltrated cells and viral particles in neuronal cytoplasm [25]. Another interesting case control study was carried out in eight public hospitals of Recife, Pernambuco state, Brazil, with neonates born during the period of January, 2016 to May, 2016. The study group was divided according to these criteria: newborns with microcephaly, defined as head circumference smaller than the average for sex and gestational age; and control group, newborns that presented no brain abnormalities. Besides that, the babies were divided into groups related to the gestational age: born at 37 weeks or more, born at 34 weeks or less and born between 34 and 36 weeks. Cerebrospinal fluid samples of the newborns and serum from the mothers were tested for African and Asian ZIKV by RT-PCR.

Results showed that 80% of the mothers had ZIKV infection, and 41% of newborns tested positive for ZIKV, evidencing the congenital ZIKV infection [26]. Further reports corroborated these findings, and the virus was already detected in placenta [27], cerebrospinal fluid [25] and retina [28] of microcephalic infants. Besides, a study with 44 women infected with ZIKV during gestation showed that the ZIKV was found in Hoffbauer cells of the placenta, and this may play a role in the dissemination of the virus during the first trimester and, thus, responsible for transferring ZIKV to the fetal brain [29]. Altogether, clinical findings were in great consonance with the fact that ZIKV is the actual etiological agent of microcephaly.

However, it was suggested that other factors could be causing microcephaly; since such correlation had never been determined for a flavivirus. For instance, some suggested that microcephaly could be caused by yellow fever vaccination during pregnancy, by the exposure to an insecticide to kill mosquito larvae, previous infection with DENV or even due to nutritional state of the mothers. Thus, at that moment, the direct causal correlation between ZIKV and microcephaly had yet to be determined, and experimental methods were the best approach.

In this context, on May 11th, 2016, three reports were published concomitantly using murine experimental models to clarify the relationship between ZIKV and microcephaly. One of the studies used the ZIKV strain isolated from French Polynesia under two experimental approaches: female C57Bl/6 IFNAR1^−^/^−^ crossed with wild type males; and wild type females treated with MAR1-5A3, an IFNAR1 antibody blocker. These models were chosen in order to facilitate viral replication, since type I interferons, as IFN-α/β, play an important role in antiviral responses, as previously demonstrated for several flaviviruses [30].

In the first approach, female mice were subcutaneously infected with 10^3^ FFU at 6.5 and 7.5 days of gestation. Analysis were performed at days P13.5 and P15.5 for viral titers, fetal body measures and cerebral histology. The pups suffered from dramatic abnormalities, such as intrauterine growth restriction (IUGR) and the presence of necrotic tissue in the placenta and brain, associated with a high rate of abortion and fetal resorption. However, there were no evident signs of microcephaly. PCR tests for ZIKV were positive in placenta and brain. On the second model, females were treated with the IFNARI blocker MAR1-5A3 at 5.5 days of gestation, infected at day 6.5 and analyzed as mentioned. IUGR was less evident and no abortion occurred. However, the presence of the virus was detected in fetal brain, and the viral titers were inversely proportional do the amount of anti-IFNRA1 used. Moreover, the research also shed light on the mechanisms of ZIKV infection on the placental compartment. Spongiotrophoblasts and glycogen trophoblasts were infected by the virus, and this was correlated with placental damage and apoptosis [30].

Another group performed the injection of ZIKV SZ01 at cerebroventricular space/lateral ventricle (LV) at day 13.5 of gestation, to circumvent mother immune response against the virus. Although this approach may not prove the vertical transmission of the virus, it may help to elucidate the mechanisms of neuronal damage. At P16.5 the presence of the virus in the brain of the pups was confirmed on the ventricular (VZ) and subventricular zones (SVZ), where most of neuronal precursor cells (NPCs) are located. Results demonstrated a significant reduction of NPCs as determined by immunofluorescence of TBR1^+^, SOX2^+^ and FOXP2^+^ cells, which co-localized active caspase-3. This was associated with reduction of the thickness of cortical plate (CP), VZ and SVZ areas of the brain, resulting in overall reduction of the brain size [31].

With the aim of identifying genes that could be master regulators for microcephaly, the group performed RNA sequencing of brain samples from infected and non-infected dams. As expected, data indicated a significant upregulation of genes involved in antiviral immune response, especially cytokines, chemokines and many interferon stimulating genes (ISGs). This is in accordance with the fact that the virus is present in the brain, and thus, eliciting a local immune response. Whether this response is mainly mounted by resident glial cells or by peripheral infiltrating leukocytes has to be determined. It was also shown that many genes involved in cell cycle were greatly altered by the presence of the virus. Thus, it is plausible to speculate that, besides inducing inflammation, this deregulation, directly or not, may greatly account for the apoptotic cell death of NPCs. The group also evaluated the expression of genes correlating directly with microcephaly, which demonstrates that many of them were downregulated. The role of these genes and phenomena orchestrated by them during ZIKV infection has still to be determined.

It is noteworthy that all the models described so far, either used IFNAR deficient animals or anti-IFNAR treatment in order to circumvent the mother innate antiviral immune response and, thus, allow virus replication and dissemination throughout the fetal body. Although these are precious and valuable approaches, they may not be ideal, as viral titers may reach very high concentration, which may not be physiological. In this sense, our group used wild-type SJL mice, which had previously shown to be susceptible to neurotropic viral infection, mainly due to their reduced production of type I interferons [32].

Thus, female SJL pregnant mice were infected intravenously with a Brazilian ZIKV isolate between days E10-E13. The findings were very consistent and corroborated by those previously mentioned [27, 28]. Pups from infected mothers presented significant IUGR, with reduced measurements for size and weight, cranial height, biparietal and crown rump length. Histological analysis was performed in several different areas of the brain, showing a reduced cortical layer thickness and intranuclear vacuolization, with chromatin margination in the cortex, thalamus and hypothalamus. No alterations were observed in cerebellum and hippocampus. This was associated with high viral titers in the brains of the pups, although it was also detected in the liver and spleen. Interestingly, we observed that infected pups also had an impaired eye formation, which was further corroborated by both experimental and clinical observations [26, 27], which will be further discussed.

In search for the mechanisms through which brain lesions were established, RNA expression targeting genes for apoptosis and autophagy was performed. Interestingly, a high increase in the expression of pro-apoptotic and autophagy genes, such as BMF, IRGM and Bcl6, was observed, supporting previous findings either in the brains of the pups or in the cultures of fibroblasts infected with ZIKV [10]. Not surprisingly, when the same set of experiments was performed with C57BL6 mice, it was clear that the virus was not able to cross the placenta and reach the fetus. PCR for ZIKV was negative and there were no macroscopic nor morphological alterations in the brain of the pups. This brings to discussion the fact that the genetics of the host is very important for the outcome of the disease. Also, it is validated by clinical observations indicating that around 30% of infected pregnant women will have babies with microcephaly [3]. In this sense, further studies on the mechanisms of resistance to ZIKV-induced congenital syndrome in human subjects need to be addressed. This kind of approach would reveal why the northeast of Brazil is still the epicenter of the epidemic and, besides, would shed light on mechanisms that may be explored by therapeutic interventions.

## ZIKV and clinical aspects

According to the WHO, children that develop microcephaly display impaired mental and intellectual ability, difficulty in motor coordination, postural balance and language. In more severe cases, they may also present seizures, epilepsy and muscle stiffness [23]. As these features are the result of the severe impairment or malformation of the brain, it is possible to correlate clinical manifestations of microcephaly children with the findings in experimental models [32]. The hypothalamus is responsible for assisting the control of the autonomous nervous system by commanding vital functions such as the respiratory and circulatory systems, body temperature, and even food intake and digestion. The thalamus is responsible for transmission of sensory impulses to the cortex, playing an important role in cognition, consciousness and control of autonomous activities. The cortex has plenty of ascending neurons responsible for memory, attention, consciousness, language, perception and thoughts. Thus, being the cortex, thalamus and hypothalamus the most affected regions during ZIKV infection, it is very understandable the presence of the aforementioned signs and symptoms in infected babies.

Several studies on the clinical aspects of the CZS have been published. A study showed anomalies observed in children of 16 pregnant women infected by ZIKV during gestation. Nine of these women had the virus in the amniotic fluid, seven had the virus in the umbilical cord blood and one in the placenta. Among these alterations, it is noticeable the loss of cerebral parenchyma volume, followed by polymicrogyria, a cortical malformation characterized by irregular and small gyration of the cortex. Moreover, anomalies in the corpus callosum like agenesia and dysgenesia, ventriculomegaly, lysencephaly, periventricular and cortical calcifications, at the junction of the white and gray mass and brainstem are present in some cases [33].

Another report with 11 newborns showed similar findings as the previous group cited, describing lisencephaly, atrophy, taquigyria, enlargement of hemisphere space and subrachnoid, shallow sulci, cortical polymicrogyria, hypoplasia of cerebellum and brain stem [34]. Histopathological findings corroborated previous observation of a gliosis outbursting pial limits with perivascular calcifications, usually associated with few infiltrating macrophages [35, 36].

One of the questions that remained unclear was if there were differences on neurological alterations among pregnant women infected in different periods of gestation. A study published in 2016 evaluated two pregnant women who had symptoms related to ZIKV infection during the 36th gestational week. The first patient was negative for TORCHES (toxoplasmosis, rubella, cytomegalovirus, herpes and syphilis) and DENV, while the second patient presented IgM and IgG for toxoplasmosis during 9th and 18th weeks of pregnancy. Besides that, both patients were positive for ZIKV in serum and urine samples. At birth, by the 38th and 39th weeks, respectively, the children were positive for ZIKV, confirming the vertical transmission, although with normal cephalic circumference and lacking ocular alterations. The transfontanellar ultrasound showed subependymal cysts and lenticulostriate vasculopathy. Although these alterations are not well understood, it indicates that ZIKV infection even during late phase of gestation may be deleterious to the babies [37]. In fact, many neurological findings have been described even in infants without microcephaly, as cortical lesions and calcifications, associated or not with cerebellar and spinal cord damage. The consequences are hyperreflexia, seizures, dysphagia, and also vision and hearing loss [38, 39]. These alterations may be more related to infection during the last trimester of gestation.

## Arthrogryposis

Although microcephaly has received major attention, ZIKV infection during pregnancy also leads to other malformations. These include mainly ocular and musculoskeletal abnormalities, such as retinal degeneration and craniofacial abnormalities with joint contractures called arthrogryposis, and in some cases, associated with spontaneous abortion [40, 41].

Arthrogryposis, first described in 1841, is defined as a congenital, non-progressive, joint contracture that affects two or more areas of the body. It is agreed that arthrogryposis is secondary to several maternal or fetal diseases, mainly those that restricts fetal movement in the uterus [42]. This reduction induces fibrosis of the joints and musculoskeletal tissues, resulting in severe limb contractures and features as clubfoot, rotated shoulder, palmar and interphalangeal contractures, arachnodactyly and several others. Although its molecular mechanisms are still under debate, genetic mutations of beta-tropomyosin, type 2 troponin, myosin heavy chain 3, myosin binding protein 1 are examples of the genetic correlation with arthrogryposis [43–45]. Moreover, neurological abnormalities of the fetus, either central – such as hydrocephaly, microcephaly, ventriculomegaly – and peripheral or neuromuscular maldevelopment, correlate to about 70–80% of the cases of arthrogryposis [46].

Either infectious or non-infectious maternal diseases may be responsible for the establishment of arthrogryposis. For example, diabetes mellitus, multiple sclerosis and myasthenia gravis or TORCH infections may greatly account [46–51]. Recently, along with microcephaly, arthrogryposis has been widely correlated with CZS. The mechanisms, however, have not been elucidated, but are likely to be consequence of the viral neurotropism, resulting in brain damage that interferes with adequate neuronal development and subsequent impaired neuromuscular signaling triggering reduction of intrauterine mobility [40, 52].

From August to October 2015, in a group of 35 infants with microcephaly related with ZIKV infection, at least 11% of them had arthrogryposis, which evidenced central or peripheral nervous system involvement. Furthermore, 25 infants (74%), had severe microcephaly and 11 (31%) presented with excessive and redundant scalp skin [53].

Martines et al. [35] presented three cases of birth followed by death of newborns whose mothers were infected with ZIKV during pregnancy. These infants had several congenital malformations, including muscle contractures, craniofacial disorders, pulmonary hypoplasia and brain abnormalities. These manifestations are characteristic of the dramatic impact of fetal ZIKV infection. Another report showed that an ultrasound test performed on a 20-year-old woman at the 18th week of gestation showed fetal weight below the mean value for gestational age. Ultrasound examinations performed at the 26th and 30th gestational week revealed microcephaly, hydranencephaly with minimal residual cortical parenchyma and, at the 32nd gestational week, undergone fetal demise. The woman passed through an induced labor and delivered a female fetus with a weight of 930 g presenting dramatic microcephaly and arthrogryposis. ZIKV was detected in extracts of cerebral cortex, medulla oblongata and cerebrospinal and amniotic fluid [54].

In another study, a Spanish group demonstrated that at 19th week of gestation, the ultrasound examination revealed fetal malformations and the ZIKV was detected in the amniotic fluid. The pregnancy was terminated at week 21. The autopsy of the fetus revealed bilateral hydrocephalus, cerebral microcalcifications and severe arthrogryposis. The skeletal muscles were underdeveloped and suffered fatty replacement, with fibrosis of the interarticular spaces. In addition, the fetus also presented hydrocephalus, dilatation of both lateral ventricles and cerebral calcifications, nevertheless, without microcephaly. ZIKV was detected in the umbilical cord and brain tissue of the fetus [55].

In summary, along with microcephaly, arthrogryposis is among the most severe features of the CZS, resulting in great impact on the lives of mothers and babies. Besides the fact that its mechanisms must be determined, there are many questions to be addressed. For example, is there a correlation between ZIKV strain, host genetics and arthrogryposis? Was there an increase in arthrogryposis in previous outbreaks, as in French Polynesia? Is there a peripheral nervous system component for the ZIKV-associated arthrogryposis?

## Ocular alterations

Ophthalmic findings in infants born from ZIKV infected mothers have been very usual in newborns with or without microcephaly during the outbreak in Brazil. It has been demonstrated that more than 80% of the infants with microcephaly examined from Recife, Bahia and São Paulo states had ophthalmoscopic abnormalities [56–58]. The first report describing ocular findings showed three children with microcephaly associated with macular pigment mottling and one of them presented a characteristic macular neuroretinal atrophy [26]. Their mothers reported symptoms such as rash and arthralgia during the first semester of pregnancy, corroborating the susceptibility period [59]. Although no real-time PCR test was performed, all TORCH infections were ruled out, and the authors declared that those cases comply criteria for ZIKV vertical transmission since cerebral calcification was detected by computed tomography scan, suggesting intrauterine infection.

Interestingly, the same group further reported ocular damage in an infant without microcephaly. Although the mother did not refer ZIKV-related symptoms during pregnancy, the newborn presented hyperreflexia at birth. Further ultrasound analysis revealed ventriculomegaly, lissencephaly and calcifications of the basal ganglia. This highlights the fact that ocular impairment is not necessarily associated with microcephaly, requiring a refined clinical evaluation [24].

Another study revealed that after retinal evaluation of 10 children with microcephaly, not only macular atrophy, but also optic nerve hypoplasia, pallor, foveal reflex loss associated with mild to moderate pigment mottling were present. As it is known that WNV can lead to macular injuries and cytomegalovirus can cause optic nerve alterations, serology tests for toxoplasmosis, rubella, cytomegalovirus, herpes simplex, syphilis and human immunodeficiency virus (HIV) were performed for all infants and the results were negative [60].

In a more substantial study, 55 infants who had mistrusted or stablished microcephaly due to presumed ZIKV congenital infection were submitted to ophthalmic assessment [61]. From the 55 children, 24 had their cerebrospinal fluid tested by IgM antibody-capture Elisa (MAC-ELISA) for ZIKV and DENV and all of them were positive for ZIKV. From the 22 remaining children, 14 presented ophthalmoscopic findings, stablishing the correlation between ZIKV and ocular findings.

Although ZIKV has been associated with ophthalmic injuries, it was still unclear if ocular alterations occurred solely in newborns with microcephaly. Ventura et al. [57], described a 57-day-old and a 6-day-old infant positive for ZIKV IgM antibody-capture Elisa in the cerebral spinal fluid associated with chorioretinal scar on the macular region and optic nerve lesion, corroborating previous findings [26, 57]. However, remarkably, microcephaly was absent in these cases [24, 62]. These reports emphasize that microcephaly is not a mandatory criterion to determine congenital ZIKV infection diagnosis, since other ZIKV-related injuries may occur.

Despite the concern with the newborns, adults may also present ocular damage associated with ZIKV infection. Non-purulent conjunctivitis and retro-orbital hyperemia are common symptoms [63]. Also, uveitis was observed in a 40-year-old man diagnosed with ZIKV [64], whose aqueous humor was positive for ZIKV RNA. Furthermore, a case of bilateral hypertensive iridocyclitis was also reported in a 39-years-old woman clinically diagnosed with ZIKV infection [65]. After the presence of classic signs of fever and rash, the patient presented bilateral ocular discomfort, blurry vision and mild redness. Very unexpectedly, a case report showed that ZIKV particles were found in the tears of a 76-year-old patient that succumbed to infection, probably due to the very high viral titers, 2.10^8^ viral particles per mL. A visiting relative swiped the tears of the patient, which was the only close contact referred, and 1 week later presented ZIKV infection symptoms, raising the question whether non-vector transmission of ZIKV is a real problem to immunocompetent hosts.

In fact, it is already well demonstrated that ZIKV may be sexually transmitted [21]. However, body fluids had never been associated with arbovirus infections before. In summary, ophthalmological alterations and injuries due to ZIKV infection are not restricted to infants, either with or without microcephaly, and may affect adults as well. These ocular findings are potential manifestations of ZIKV infection and the symptoms should not be neglected [66].

The mechanisms through which ZIKV causes ocular damage have started to be elucidated in experimental models. Through the infection of pregnant IFNARI deficient mice or wild type mice treated with anti-IFNARI monoclonal antibodies, it was demonstrated that the virus targets the retina, iris and optic nerve causing panuveitis and the shedding of viral particles in the tears at 3.10^2^ FFUs/mL. Even after 28 days of infection and clearance of the virus in the serum, ZIKV was still detected in the eye and tears. Infectivity of the particles were confirmed after inoculation with eye homogenates in AG129 mice, which presented ocular pathology, demonstrating that the virus present in the eye remains infectious. This group also tested the prevalence of the virus in the eyes of congenitally infected offspring but only 5% of eyes remained positive for viral RNA [67].

These findings suggest that the eye can support viral replication 3 weeks after infection, which brings to attention a different non-vector transmission of ZIKV. Nevertheless, the mechanism of eye infection remains uncertain. Double knockout mice to the receptors previously described as required to ZIKV infection showed no difference levels of ZIKV present in the eyes and other organs [27, 40]. Thus, the experimental models developed are essential to elucidate the path through which ZIKV reaches the eyes and causes damage [38].

## Conclusion

ZIKV epidemics has reminded us the fragility of the human beings to emerging infectious diseases, as previously experienced with many other agents. Moreover, ZIKV also changed the way researchers and physicians deal with flavivirus infections. This is due mainly to the severe impact of ZIKV infection during pregnancy and the resulting CZS, with microcephaly, arthrogryposis and retinal damage (Table 1). Moreover, it may be sexually transmitted, which has never been observed for a flavivirus. Researchers have a long road to a better understanding of the molecular and cellular mechanisms behind the CZS and to the development of effective therapeutic interventions or vaccine approaches. These must be considered a priority, not only to stop the spreading of the virus and the dramatic impact of microcephaly, but also to prepare us for further epidemics.

**Table 1:** Clinical aspects of newborns from infected mothers with Zika virus during pregnancy

Abnormalities	References
Brain	
Ventriculomegaly	[3, 24, 34]
Cortical and subcortical calcifications	[3, 24, 34, 36, 37, 39, 40, 56]
Agyria, taquigyria or polymicrogyria	[3, 34, 35]
Collapsed or dilated ventricle	[3, 56]
Agenesia	[34]
Lysencephaly	[24, 34]
Hypoplasia of cerebellum	[35]
Hydranencephaly	[55, 56]
Loss of cerebral parenchyma volume	[34]
Astroglyosis	[3]
Ocular	
Uveitis	[24]
Retinal degeneration	[24]
Neuroretinal atrophy	[28]
Macular atrophy	[60]
Optic nerve hypoplasia	[58, 60]
Macular pigment mottling	[28]
Chorioretinal scar	[28, 58]
Others	
Arthrogryposis	[54–56]
Intrauterine growth restriction	[3]
Congenital contractures	[36]
Pulmonary hypoplasia	[36]
Craniofacial malformations	[36]

## Abbreviations

C: Capside
CHIKV: Chikungunya vírus
CP: Cortical plate
CZS: Congenital Zika syndrome
DENV: Dengue virus
Env: Envelope
GBS: Guillain-Barré syndrome
HIV: Human immunodeficiency virus
IFNAR1: Interferon alfa receptor
ISGs: Interferon stimulating genes
IURG: Intrauterine growth restriction
JEV: Japanese Encephalytis
LV: Lateral ventricle
MAC-ELISA: IgM antibody-capture Elisa
NPCs: Neuronal precursor cells
NS1, NS2a-2b, NS3, NS4a-4b, NS5: Non-structural proteins
PHEIC: Public Health Emergency of International Concern
Pr-M: Pre-membrane
SVZ: Subventricular zone
TORCHES: Toxoplasmosis, rubella, cytomegalovirus, herpes and syphilis
VZ: Ventricular zone
WHO: World Health Organization
WNV: West Nile virus
YFV: Yellow fever virus
ZIKV: Zika virus
